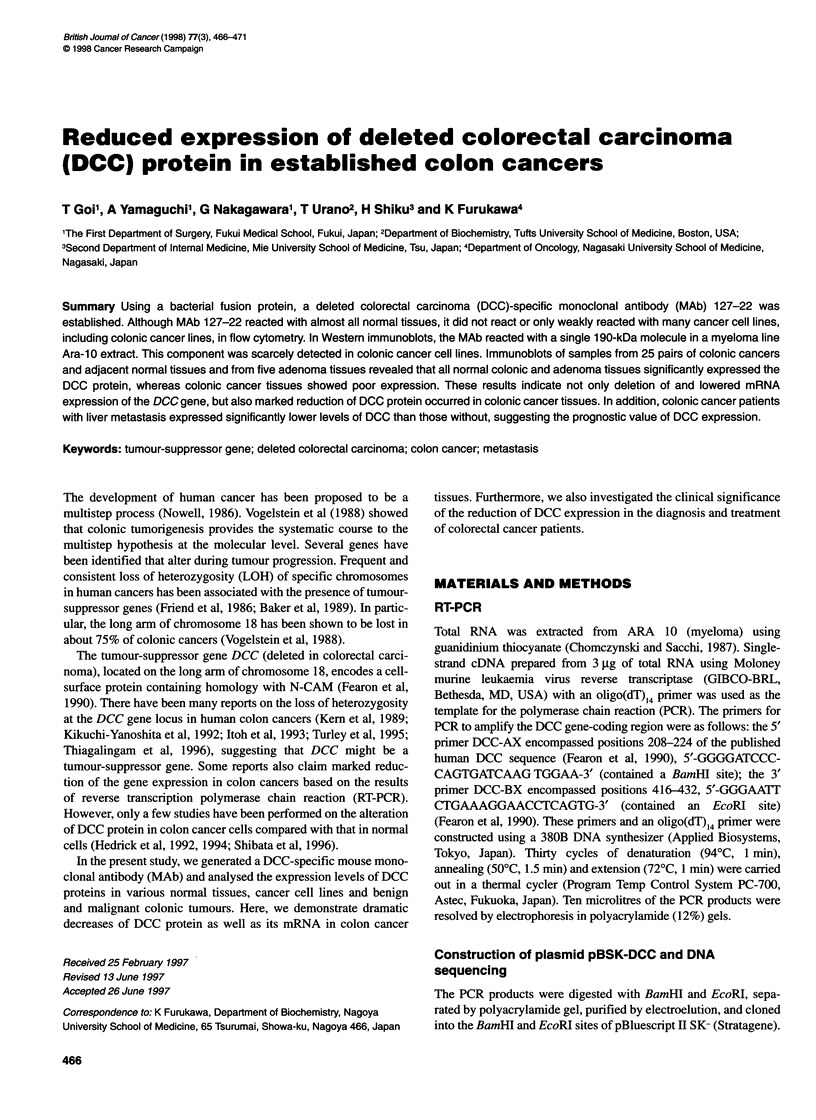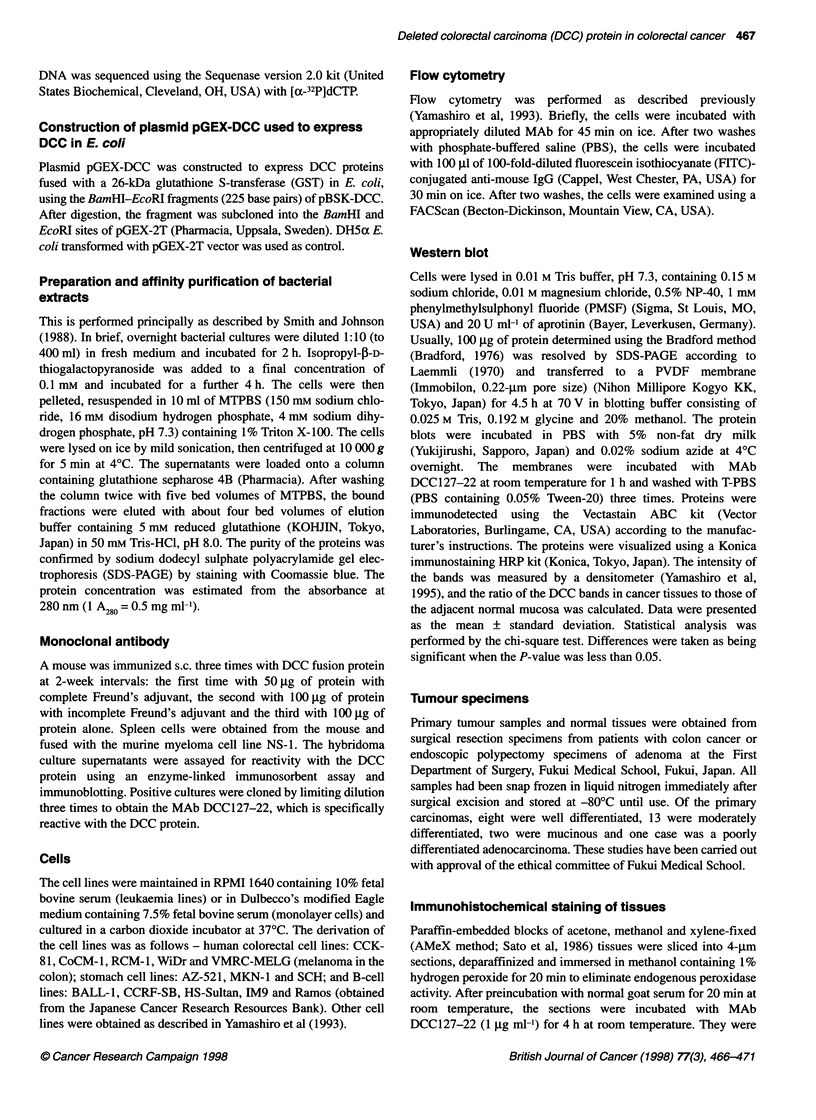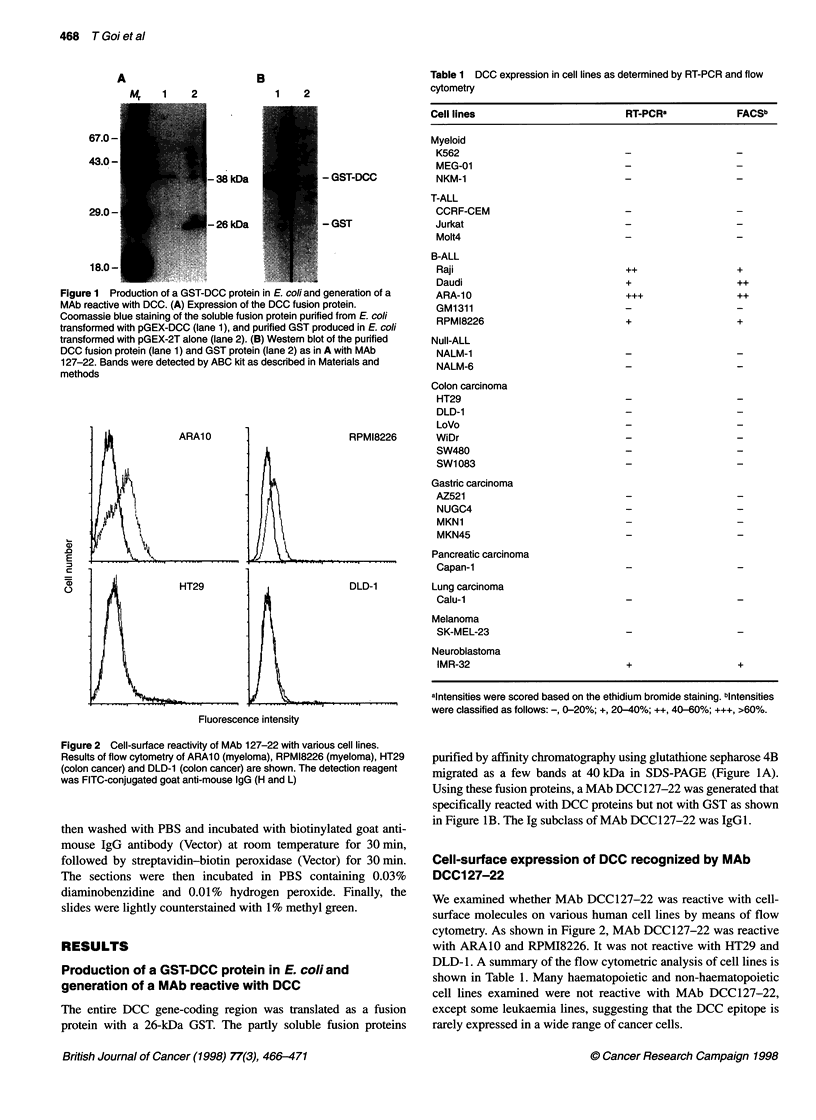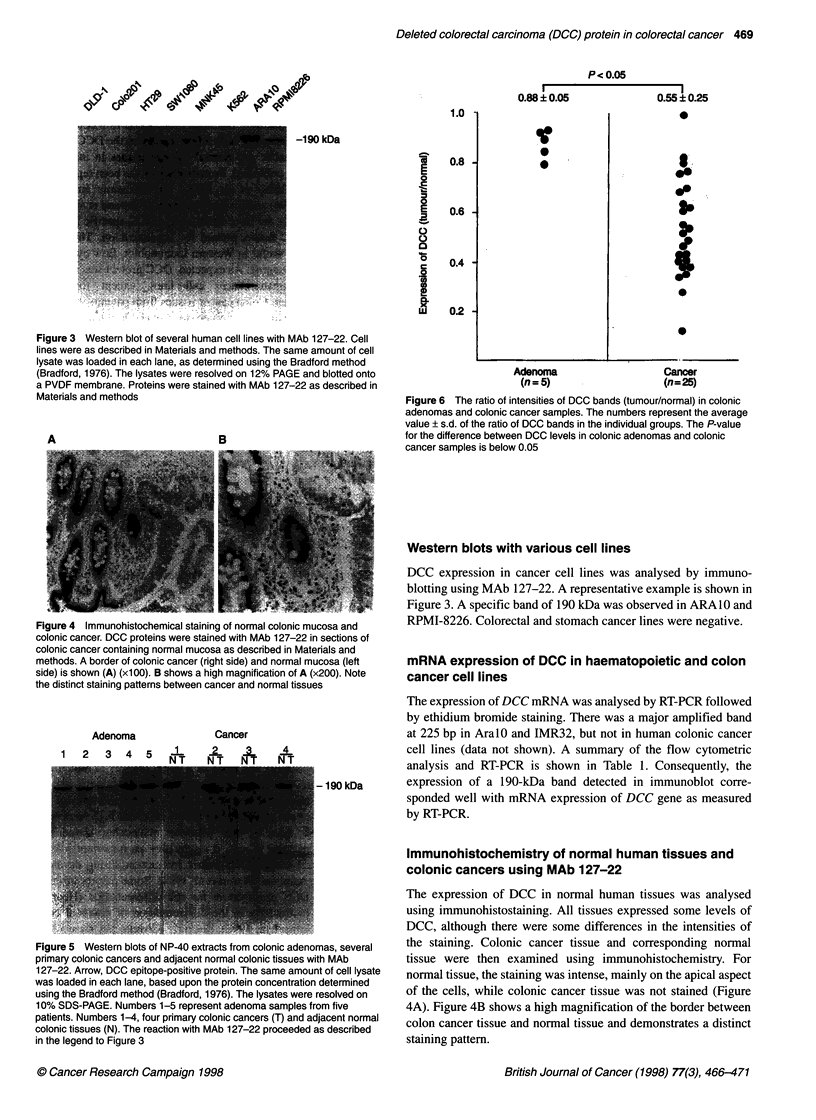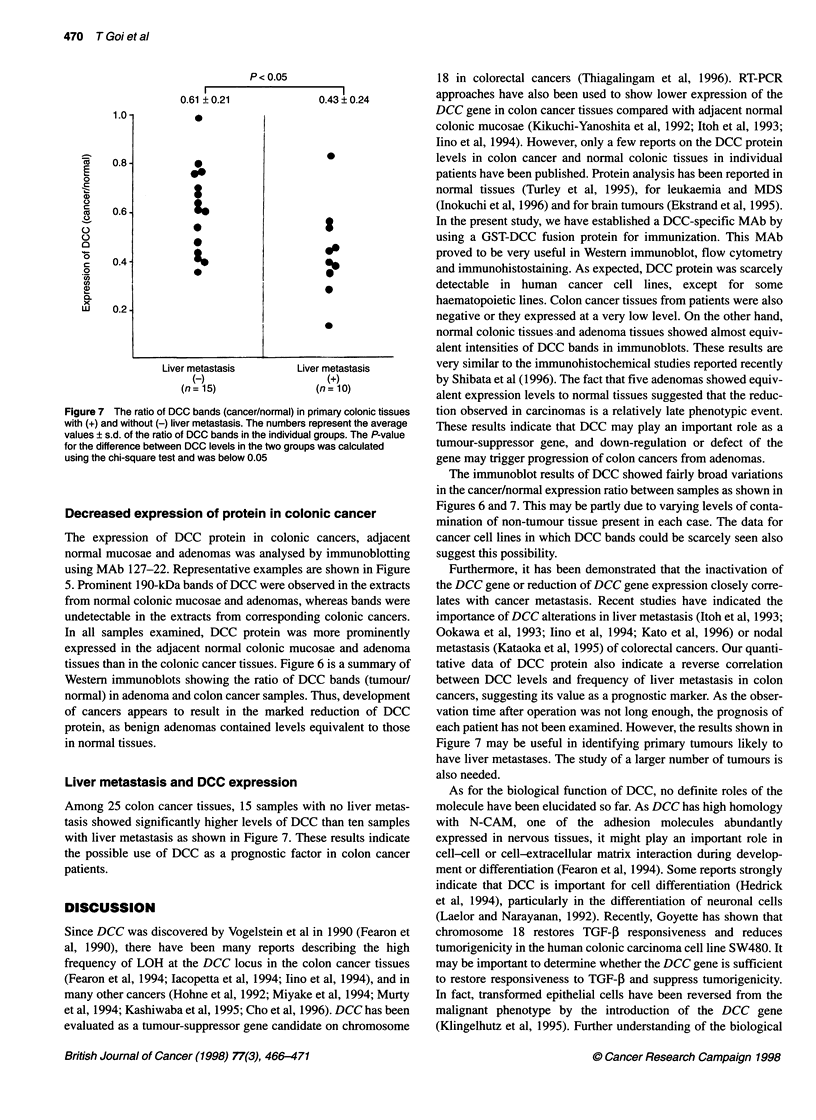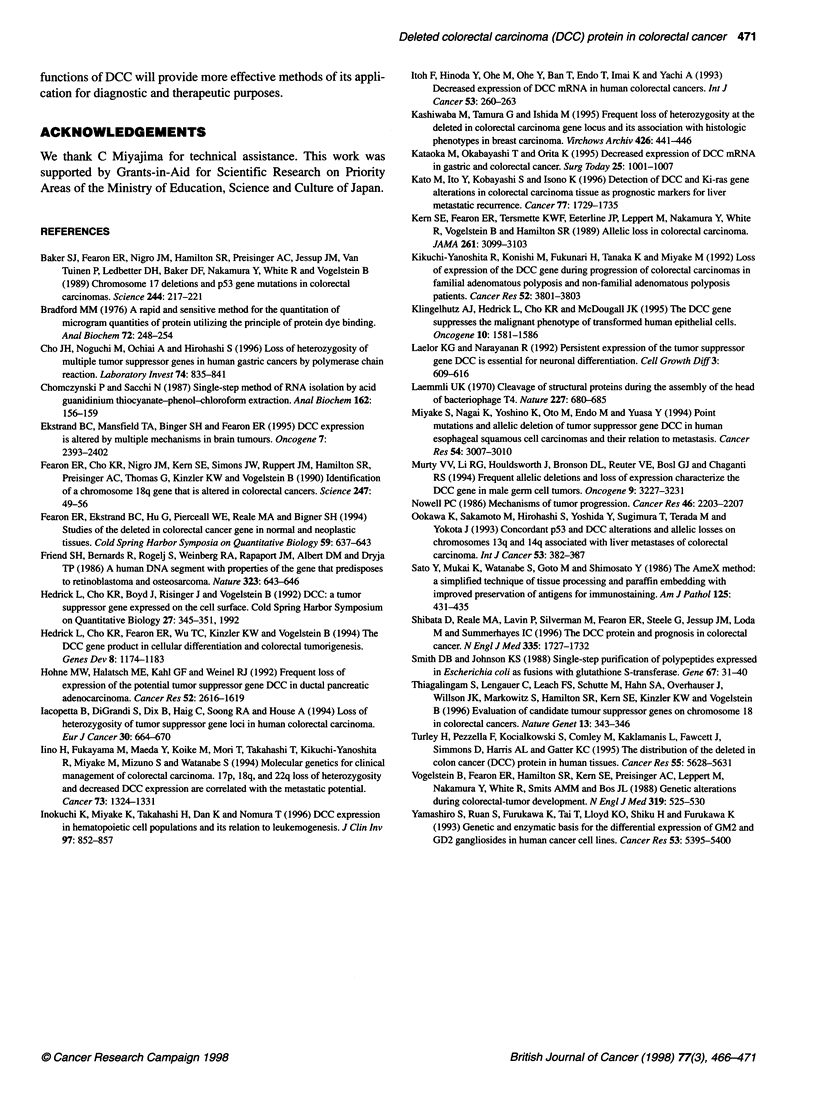# Reduced expression of deleted colorectal carcinoma (DCC) protein in established colon cancers.

**DOI:** 10.1038/bjc.1998.74

**Published:** 1998

**Authors:** T. Goi, A. Yamaguchi, G. Nakagawara, T. Urano, H. Shiku, K. Furukawa

**Affiliations:** The First Department of Surgery, Fukui Medical School, Japan.

## Abstract

**Images:**


British Journal of Cancer (1998) 77(3), 466–471
© 1998 Cancer Research Campaign

### Reduced expression of deleted colorectal carcinoma (DCC) protein in established colon cancers

**T Goi[1], A Yamaguchi[1], G Nakagawara[1], T Urano[2], H Shiku[3] and K Furukawa[4]**

[1]The First Department of Surgery, Fukui Medical School, Fukui, Japan; [2]Department of Biochemistry, Tufts University School of Medicine, Boston, USA; [3]Second Department of Internal Medicine, Mie University School of Medicine, Tsu, Japan; [4]Department of Oncology, Nagasaki University School of Medicine, Nagasaki, Japan

**Summary** Using a bacterial fusion protein, a deleted colorectal carcinoma (DCC)-specific monoclonal antibody (MAb) 127–22 was established. Although MAb 127–22 reacted with almost all normal tissues, it did not react or only weakly reacted with many cancer cell lines, including colonic cancer lines, in flow cytometry. In Western immunoblots, the MAb reacted with a single 190-kDa molecule in a myeloma line Ara-10 extract. This component was scarcely detected in colonic cancer cell lines. Immunoblots of samples from 25 pairs of colonic cancers and adjacent normal tissues and from five adenoma tissues revealed that all normal colonic and adenoma tissues significantly expressed the DCC protein, whereas colonic cancer tissues showed poor expression. These results indicate not only deletion of and lowered mRNA expression of the *DCC* gene, but also marked reduction of DCC protein occurred in colonic cancer tissues. In addition, colonic cancer patients with liver metastasis expressed significantly lower levels of DCC than those without, suggesting the prognostic value of DCC expression.

**Keywords:** tumour-suppressor gene; deleted colorectal carcinoma; colon cancer; metastasis

The development of human cancer has been proposed to be a multistep process (Nowell, 1986). Vogelstein et al (1988) showed that colonic tumorigenesis provides the systematic course to the multistep hypothesis at the molecular level. Several genes have been identified that alter during tumour progression. Frequent and consistent loss of heterozygosity (LOH) of specific chromosomes in human cancers has been associated with the presence of tumour-suppressor genes (Friend et al, 1986; Baker et al, 1989). In particular, the long arm of chromosome 18 has been shown to be lost in about 75% of colonic cancers (Vogelstein et al, 1988).

The tumour-suppressor gene *DCC* (deleted in colorectal carcinoma), located on the long arm of chromosome 18, encodes a cell-surface protein containing homology with N-CAM (Fearon et al, 1990). There have been many reports on the loss of heterozygosity at the *DCC* gene locus in human colon cancers (Kern et al, 1989; Kikuchi-Yanoshita et al, 1992; Itoh et al, 1993; Turley et al, 1995; Thiagalingam et al, 1996), suggesting that *DCC* might be a tumour-suppressor gene. Some reports also claim marked reduction of the gene expression in colon cancers based on the results of reverse transcription polymerase chain reaction (RT-PCR). However, only a few studies have been performed on the alteration of DCC protein in colon cancer cells compared with that in normal cells (Hedrick et al, 1992, 1994; Shibata et al, 1996).

In the present study, we generated a DCC-specific mouse monoclonal antibody (MAb) and analysed the expression levels of DCC proteins in various normal tissues, cancer cell lines and benign and malignant colonic tumours. Here, we demonstrate dramatic decreases of DCC protein as well as its mRNA in colon cancer tissues. Furthermore, we also investigated the clinical significance of the reduction of DCC expression in the diagnosis and treatment of colorectal cancer patients.

*Received 25 February 1997*
*Revised 13 June 1997*
*Accepted 26 June 1997*

*Correspondence to:* K Furukawa, Department of Biochemistry, Nagoya University School of Medicine, 65 Tsurumai, Showa-ku, Nagoya 466, Japan

### MATERIALS AND METHODS

### RT-PCR

Total RNA was extracted from ARA 10 (myeloma) using guanidinium thiocyanate (Chomczynski and Sacchi, 1987). Single-strand cDNA prepared from 3 μg of total RNA using Moloney murine leukaemia virus reverse transcriptase (GIBCO-BRL, Bethesda, MD, USA) with an oligo$(dT)_{14}$ primer was used as the template for the polymerase chain reaction (PCR). The primers for PCR to amplify the DCC gene-coding region were as follows: the 5′ primer DCC-AX encompassed positions 208–224 of the published human DCC sequence (Fearon et al, 1990), 5′-GGGGATCCC-CAGTGATCAAG TGGAA-3′ (contained a *Bam*HI site); the 3′ primer DCC-BX encompassed positions 416–432, 5′-GGGAATT CTGAAAGGAACCTCAGTG-3′ (contained an *Eco*RI site) (Fearon et al, 1990). These primers and an oligo$(dT)_{14}$ primer were constructed using a 380B DNA synthesizer (Applied Biosystems, Tokyo, Japan). Thirty cycles of denaturation (94°C, 1 min), annealing (50°C, 1.5 min) and extension (72°C, 1 min) were carried out in a thermal cycler (Program Temp Control System PC-700, Astec, Fukuoka, Japan). Ten microlitres of the PCR products were resolved by electrophoresis in polyacrylamide (12%) gels.

### Construction of plasmid pBSK-DCC and DNA sequencing

The PCR products were digested with *Bam*HI and *Eco*RI, separated by polyacrylamide gel, purified by electroelution, and cloned into the *Bam*HI and *Eco*RI sites of pBluescript II SK$^-$ (Stratagene).

DNA was sequenced using the Sequenase version 2.0 kit (United States Biochemical, Cleveland, OH, USA) with [$\alpha$-$^{32}$P]dCTP.

### Construction of plasmid pGEX-DCC used to express DCC in *E. coli*

Plasmid pGEX-DCC was constructed to express DCC proteins fused with a 26-kDa glutathione S-transferase (GST) in *E. coli*, using the *Bam*HI–*Eco*RI fragments (225 base pairs) of pBSK-DCC. After digestion, the fragment was subcloned into the *Bam*HI and *Eco*RI sites of pGEX-2T (Pharmacia, Uppsala, Sweden). DH5$\alpha$ *E. coli* transformed with pGEX-2T vector was used as control.

### Preparation and affinity purification of bacterial extracts

This is performed principally as described by Smith and Johnson (1988). In brief, overnight bacterial cultures were diluted 1:10 (to 400 ml) in fresh medium and incubated for 2 h. Isopropyl-$\beta$-D-thiogalactopyranoside was added to a final concentration of 0.1 mM and incubated for a further 4 h. The cells were then pelleted, resuspended in 10 ml of MTPBS (150 mM sodium chloride, 16 mM disodium hydrogen phosphate, 4 mM sodium dihydrogen phosphate, pH 7.3) containing 1% Triton X-100. The cells were lysed on ice by mild sonication, then centrifuged at 10 000 *g* for 5 min at 4°C. The supernatants were loaded onto a column containing glutathione sepharose 4B (Pharmacia). After washing the column twice with five bed volumes of MTPBS, the bound fractions were eluted with about four bed volumes of elution buffer containing 5 mM reduced glutathione (KOHJIN, Tokyo, Japan) in 50 mM Tris-HCl, pH 8.0. The purity of the proteins was confirmed by sodium dodecyl sulphate polyacrylamide gel electrophoresis (SDS-PAGE) by staining with Coomassie blue. The protein concentration was estimated from the absorbance at 280 nm (1 $A_{280}$ = 0.5 mg ml$^{-1}$).

### Monoclonal antibody

A mouse was immunized s.c. three times with DCC fusion protein at 2-week intervals: the first time with 50 µg of protein with complete Freund's adjuvant, the second with 100 µg of protein with incomplete Freund's adjuvant and the third with 100 µg of protein alone. Spleen cells were obtained from the mouse and fused with the murine myeloma cell line NS-1. The hybridoma culture supernatants were assayed for reactivity with the DCC protein using an enzyme-linked immunosorbent assay and immunoblotting. Positive cultures were cloned by limiting dilution three times to obtain the MAb DCC127–22, which is specifically reactive with the DCC protein.

### Cells

The cell lines were maintained in RPMI 1640 containing 10% fetal bovine serum (leukaemia lines) or in Dulbecco's modified Eagle medium containing 7.5% fetal bovine serum (monolayer cells) and cultured in a carbon dioxide incubator at 37°C. The derivation of the cell lines was as follows – human colorectal cell lines: CCK-81, CoCM-1, RCM-1, WiDr and VMRC-MELG (melanoma in the colon); stomach cell lines: AZ-521, MKN-1 and SCH; and B-cell lines: BALL-1, CCRF-SB, HS-Sultan, IM9 and Ramos (obtained from the Japanese Cancer Research Resources Bank). Other cell lines were obtained as described in Yamashiro et al (1993).

### Flow cytometry

Flow cytometry was performed as described previously (Yamashiro et al, 1993). Briefly, the cells were incubated with appropriately diluted MAb for 45 min on ice. After two washes with phosphate-buffered saline (PBS), the cells were incubated with 100 µl of 100-fold-diluted fluorescein isothiocyanate (FITC)-conjugated anti-mouse IgG (Cappel, West Chester, PA, USA) for 30 min on ice. After two washes, the cells were examined using a FACScan (Becton-Dickinson, Mountain View, CA, USA).

### Western blot

Cells were lysed in 0.01 M Tris buffer, pH 7.3, containing 0.15 M sodium chloride, 0.01 M magnesium chloride, 0.5% NP-40, 1 mM phenylmethylsulphonyl fluoride (PMSF) (Sigma, St Louis, MO, USA) and 20 U ml$^{-1}$ of aprotinin (Bayer, Leverkusen, Germany). Usually, 100 µg of protein determined using the Bradford method (Bradford, 1976) was resolved by SDS-PAGE according to Laemmli (1970) and transferred to a PVDF membrane (Immobilon, 0.22-µm pore size) (Nihon Millipore Kogyo KK, Tokyo, Japan) for 4.5 h at 70 V in blotting buffer consisting of 0.025 M Tris, 0.192 M glycine and 20% methanol. The protein blots were incubated in PBS with 5% non-fat dry milk (Yukijirushi, Sapporo, Japan) and 0.02% sodium azide at 4°C overnight. The membranes were incubated with MAb DCC127–22 at room temperature for 1 h and washed with T-PBS (PBS containing 0.05% Tween-20) three times. Proteins were immunodetected using the Vectastain ABC kit (Vector Laboratories, Burlingame, CA, USA) according to the manufacturer's instructions. The proteins were visualized using a Konica immunostaining HRP kit (Konica, Tokyo, Japan). The intensity of the bands was measured by a densitometer (Yamashiro et al, 1995), and the ratio of the DCC bands in cancer tissues to those of the adjacent normal mucosa was calculated. Data were presented as the mean ± standard deviation. Statistical analysis was performed by the chi-square test. Differences were taken as being significant when the *P*-value was less than 0.05.

### Tumour specimens

Primary tumour samples and normal tissues were obtained from surgical resection specimens from patients with colon cancer or endoscopic polypectomy specimens of adenoma at the First Department of Surgery, Fukui Medical School, Fukui, Japan. All samples had been snap frozen in liquid nitrogen immediately after surgical excision and stored at –80°C until use. Of the primary carcinomas, eight were well differentiated, 13 were moderately differentiated, two were mucinous and one case was a poorly differentiated adenocarcinoma. These studies have been carried out with approval of the ethical committee of Fukui Medical School.

### Immunohistochemical staining of tissues

Paraffin-embedded blocks of acetone, methanol and xylene-fixed (AMeX method; Sato et al, 1986) tissues were sliced into 4-µm sections, deparaffinized and immersed in methanol containing 1% hydrogen peroxide for 20 min to eliminate endogenous peroxidase activity. After preincubation with normal goat serum for 20 min at room temperature, the sections were incubated with MAb DCC127–22 (1 µg ml$^{-1}$) for 4 h at room temperature. They were

© Cancer Research Campaign 1998

**Figure 1** Production of a GST-DCC protein in *E. coli* and generation of a MAb reactive with DCC. (**A**) Expression of the DCC fusion protein. Coomassie blue staining of the soluble fusion protein purified from *E. coli* transformed with pGEX-DCC (lane 1), and purified GST produced in *E. coli* transformed with pGEX-2T alone (lane 2). (**B**) Western blot of the purified DCC fusion protein (lane 1) and GST protein (lane 2) as in **A** with MAb 127–22. Bands were detected by ABC kit as described in Materials and methods

**Figure 2** Cell-surface reactivity of MAb 127–22 with various cell lines. Results of flow cytometry of ARA10 (myeloma), RPMI8226 (myeloma), HT29 (colon cancer) and DLD-1 (colon cancer) are shown. The detection reagent was FITC-conjugated goat anti-mouse IgG (H and L)

then washed with PBS and incubated with biotinylated goat anti-mouse IgG antibody (Vector) at room temperature for 30 min, followed by streptavidin–biotin peroxidase (Vector) for 30 min. The sections were then incubated in PBS containing 0.03% diaminobenzidine and 0.01% hydrogen peroxide. Finally, the slides were lightly counterstained with 1% methyl green.

### RESULTS

### Production of a GST-DCC protein in *E. coli* and generation of a MAb reactive with DCC

The entire DCC gene-coding region was translated as a fusion protein with a 26-kDa GST. The partly soluble fusion proteins

**Table 1** DCC expression in cell lines as determined by RT-PCR and flow cytometry

| Cell lines | RT-PCR[a] | FACS[b] |
|---|---|---|
| Myeloid | | |
| K562 | – | – |
| MEG-01 | – | – |
| NKM-1 | – | – |
| T-ALL | | |
| CCRF-CEM | – | – |
| Jurkat | – | – |
| Molt4 | – | – |
| B-ALL | | |
| Raji | ++ | + |
| Daudi | + | ++ |
| ARA-10 | +++ | ++ |
| GM1311 | – | – |
| RPMI8226 | + | + |
| Null-ALL | | |
| NALM-1 | – | – |
| NALM-6 | – | – |
| Colon carcinoma | | |
| HT29 | – | – |
| DLD-1 | – | – |
| LoVo | – | – |
| WiDr | – | – |
| SW480 | – | – |
| SW1083 | – | – |
| Gastric carcinoma | | |
| AZ521 | – | – |
| NUGC4 | – | – |
| MKN1 | – | – |
| MKN45 | – | – |
| Pancreatic carcinoma | | |
| Capan-1 | – | – |
| Lung carcinoma | | |
| Calu-1 | – | – |
| Melanoma | | |
| SK-MEL-23 | – | – |
| Neuroblastoma | | |
| IMR-32 | + | + |

[a]Intensities were scored based on the ethidium bromide staining. [b]Intensities were classified as follows: –, 0–20%; +, 20–40%; ++, 40–60%; +++, >60%.

purified by affinity chromatography using glutathione sepharose 4B migrated as a few bands at 40 kDa in SDS-PAGE (Figure 1A). Using these fusion proteins, a MAb DCC127–22 was generated that specifically reacted with DCC proteins but not with GST as shown in Figure 1B. The Ig subclass of MAb DCC127–22 was IgG1.

### Cell-surface expression of DCC recognized by MAb DCC127–22

We examined whether MAb DCC127–22 was reactive with cell-surface molecules on various human cell lines by means of flow cytometry. As shown in Figure 2, MAb DCC127–22 was reactive with ARA10 and RPMI8226. It was not reactive with HT29 and DLD-1. A summary of the flow cytometric analysis of cell lines is shown in Table 1. Many haematopoietic and non-haematopoietic cell lines examined were not reactive with MAb DCC127–22, except some leukaemia lines, suggesting that the DCC epitope is rarely expressed in a wide range of cancer cells.

 © Cancer Research Campaign 1998

**Figure 3** Western blot of several human cell lines with MAb 127–22. Cell lines were as described in Materials and methods. The same amount of cell lysate was loaded in each lane, as determined using the Bradford method (Bradford, 1976). The lysates were resolved on 12% PAGE and blotted onto a PVDF membrane. Proteins were stained with MAb 127–22 as described in Materials and methods

**Figure 4** Immunohistochemical staining of normal colonic mucosa and colonic cancer. DCC proteins were stained with MAb 127–22 in sections of colonic cancer containing normal mucosa as described in Materials and methods. A border of colonic cancer (right side) and normal mucosa (left side) is shown (**A**) (×100). **B** shows a high magnification of **A** (×200). Note the distinct staining patterns between cancer and normal tissues

**Figure 5** Western blots of NP-40 extracts from colonic adenomas, several primary colonic cancers and adjacent normal colonic tissues with MAb 127–22. Arrow, DCC epitope-positive protein. The same amount of cell lysate was loaded in each lane, based upon the protein concentration determined using the Bradford method (Bradford, 1976). The lysates were resolved on 10% SDS-PAGE. Numbers 1–5 represent adenoma samples from five patients. Numbers 1–4, four primary colonic cancers (T) and adjacent normal colonic tissues (N). The reaction with MAb 127–22 proceeded as described in the legend to Figure 3

**Figure 6** The ratio of intensities of DCC bands (tumour/normal) in colonic adenomas and colonic cancer samples. The numbers represent the average value ± s.d. of the ratio of DCC bands in the individual groups. The *P*-value for the difference between DCC levels in colonic adenomas and colonic cancer samples is below 0.05

### Western blots with various cell lines

DCC expression in cancer cell lines was analysed by immunoblotting using MAb 127–22. A representative example is shown in Figure 3. A specific band of 190 kDa was observed in ARA10 and RPMI-8226. Colorectal and stomach cancer lines were negative.

### mRNA expression of DCC in haematopoietic and colon cancer cell lines

The expression of *DCC* mRNA was analysed by RT-PCR followed by ethidium bromide staining. There was a major amplified band at 225 bp in Ara10 and IMR32, but not in human colonic cancer cell lines (data not shown). A summary of the flow cytometric analysis and RT-PCR is shown in Table 1. Consequently, the expression of a 190-kDa band detected in immunoblot corresponded well with mRNA expression of *DCC* gene as measured by RT-PCR.

### Immunohistochemistry of normal human tissues and colonic cancers using MAb 127–22

The expression of DCC in normal human tissues was analysed using immunohistostaining. All tissues expressed some levels of DCC, although there were some differences in the intensities of the staining. Colonic cancer tissue and corresponding normal tissue were then examined using immunohistochemistry. For normal tissue, the staining was intense, mainly on the apical aspect of the cells, while colonic cancer tissue was not stained (Figure 4A). Figure 4B shows a high magnification of the border between colon cancer tissue and normal tissue and demonstrates a distinct staining pattern.

**Figure 7** The ratio of DCC bands (cancer/normal) in primary colonic tissues with (+) and without (–) liver metastasis. The numbers represent the average values ± s.d. of the ratio of DCC bands in the individual groups. The *P*-value for the difference between DCC levels in the two groups was calculated using the chi-square test and was below 0.05

### Decreased expression of protein in colonic cancer

The expression of DCC protein in colonic cancers, adjacent normal mucosae and adenomas was analysed by immunoblotting using MAb 127–22. Representative examples are shown in Figure 5. Prominent 190-kDa bands of DCC were observed in the extracts from normal colonic mucosae and adenomas, whereas bands were undetectable in the extracts from corresponding colonic cancers. In all samples examined, DCC protein was more prominently expressed in the adjacent normal colonic mucosae and adenoma tissues than in the colonic cancer tissues. Figure 6 is a summary of Western immunoblots showing the ratio of DCC bands (tumour/normal) in adenoma and colon cancer samples. Thus, development of cancers appears to result in the marked reduction of DCC protein, as benign adenomas contained levels equivalent to those in normal tissues.

### Liver metastasis and DCC expression

Among 25 colon cancer tissues, 15 samples with no liver metastasis showed significantly higher levels of DCC than ten samples with liver metastasis as shown in Figure 7. These results indicate the possible use of DCC as a prognostic factor in colon cancer patients.

### DISCUSSION

Since *DCC* was discovered by Vogelstein et al in 1990 (Fearon et al, 1990), there have been many reports describing the high frequency of LOH at the *DCC* locus in the colon cancer tissues (Fearon et al, 1994; Iacopetta et al, 1994; Iino et al, 1994), and in many other cancers (Hohne et al, 1992; Miyake et al, 1994; Murty et al, 1994; Kashiwaba et al, 1995; Cho et al, 1996). *DCC* has been evaluated as a tumour-suppressor gene candidate on chromosome 18 in colorectal cancers (Thiagalingam et al, 1996). RT-PCR approaches have also been used to show lower expression of the *DCC* gene in colon cancer tissues compared with adjacent normal colonic mucosae (Kikuchi-Yanoshita et al, 1992; Itoh et al, 1993; Iino et al, 1994). However, only a few reports on the DCC protein levels in colon cancer and normal colonic tissues in individual patients have been published. Protein analysis has been reported in normal tissues (Turley et al, 1995), for leukaemia and MDS (Inokuchi et al, 1996) and for brain tumours (Ekstrand et al, 1995). In the present study, we have established a DCC-specific MAb by using a GST-DCC fusion protein for immunization. This MAb proved to be very useful in Western immunoblot, flow cytometry and immunohistostaining. As expected, DCC protein was scarcely detectable in human cancer cell lines, except for some haematopoietic lines. Colon cancer tissues from patients were also negative or they expressed at a very low level. On the other hand, normal colonic tissues and adenoma tissues showed almost equivalent intensities of DCC bands in immunoblots. These results are very similar to the immunohistochemical studies reported recently by Shibata et al (1996). The fact that five adenomas showed equivalent expression levels to normal tissues suggested that the reduction observed in carcinomas is a relatively late phenotypic event. These results indicate that DCC may play an important role as a tumour-suppressor gene, and down-regulation or defect of the gene may trigger progression of colon cancers from adenomas.

The immunoblot results of DCC showed fairly broad variations in the cancer/normal expression ratio between samples as shown in Figures 6 and 7. This may be partly due to varying levels of contamination of non-tumour tissue present in each case. The data for cancer cell lines in which DCC bands could be scarcely seen also suggest this possibility.

Furthermore, it has been demonstrated that the inactivation of the *DCC* gene or reduction of *DCC* gene expression closely correlates with cancer metastasis. Recent studies have indicated the importance of *DCC* alterations in liver metastasis (Itoh et al, 1993; Ookawa et al, 1993; Iino et al, 1994; Kato et al, 1996) or nodal metastasis (Kataoka et al, 1995) of colorectal cancers. Our quantitative data of DCC protein also indicate a reverse correlation between DCC levels and frequency of liver metastasis in colon cancers, suggesting its value as a prognostic marker. As the observation time after operation was not long enough, the prognosis of each patient has not been examined. However, the results shown in Figure 7 may be useful in identifying primary tumours likely to have liver metastases. The study of a larger number of tumours is also needed.

As for the biological function of DCC, no definite roles of the molecule have been elucidated so far. As *DCC* has high homology with N-CAM, one of the adhesion molecules abundantly expressed in nervous tissues, it might play an important role in cell–cell or cell–extracellular matrix interaction during development or differentiation (Fearon et al, 1994). Some reports strongly indicate that DCC is important for cell differentiation (Hedrick et al, 1994), particularly in the differentiation of neuronal cells (Laelor and Narayanan, 1992). Recently, Goyette has shown that chromosome 18 restores TGF-β responsiveness and reduces tumorigenicity in the human colonic carcinoma cell line SW480. It may be important to determine whether the *DCC* gene is sufficient to restore responsiveness to TGF-β and suppress tumorigenicity. In fact, transformed epithelial cells have been reversed from the malignant phenotype by the introduction of the *DCC* gene (Klingelhutz et al, 1995). Further understanding of the biological

© Cancer Research Campaign 1998

functions of DCC will provide more effective methods of its application for diagnostic and therapeutic purposes.

### ACKNOWLEDGEMENTS

We thank C Miyajima for technical assistance. This work was supported by Grants-in-Aid for Scientific Research on Priority Areas of the Ministry of Education, Science and Culture of Japan.

### REFERENCES

Baker SJ, Fearon ER, Nigro JM, Hamilton SR, Preisinger AC, Jessup JM, Van Tuinen P, Ledbetter DH, Baker DF, Nakamura Y, White R and Vogelstein B (1989) Chromosome 17 deletions and p53 gene mutations in colorectal carcinomas. *Science* **244**: 217–221

Bradford MM (1976) A rapid and sensitive method for the quantitation of microgram quantities of protein utilizing the principle of protein dye binding. *Anal Biochem* **72**: 248–254

Cho JH, Noguchi M, Ochiai A and Hirohashi S (1996) Loss of heterozygosity of multiple tumor suppressor genes in human gastric cancers by polymerase chain reaction. *Laboratory Invest* **74**: 835–841

Chomczynski P and Sacchi N (1987) Single-step method of RNA isolation by acid guanidinium thiocyanate–phenol–chloroform extraction. *Anal Biochem* **162**: 156–159

Ekstrand BC, Mansfield TA, Binger SH and Fearon ER (1995) DCC expression is altered by multiple mechanisms in brain tumours. *Oncogene* **7**: 2393–2402

Fearon ER, Cho KR, Nigro JM, Kern SE, Simons JW, Ruppert JM, Hamilton SR, Preisinger AC, Thomas G, Kinzler KW and Vogelstein B (1990) Identification of a chromosome 18q gene that is altered in colorectal cancers. *Science* **247**: 49–56

Fearon ER, Ekstrand BC, Hu G, Pierceall WE, Reale MA and Bigner SH (1994) Studies of the deleted in colorectal cancer gene in normal and neoplastic tissues. *Cold Spring Harbor Symposia on Quantitative Biology* **59**: 637–643

Friend SH, Bernards R, Rogelj S, Weinberg RA, Rapaport JM, Albert DM and Dryja TP (1986) A human DNA segment with properties of the gene that predisposes to retinoblastoma and osteosarcoma. *Nature* **323**: 643–646

Hedrick L, Cho KR, Boyd J, Risinger J and Vogelstein B (1992) DCC: a tumor suppressor gene expressed on the cell surface. Cold Spring Harbor Symposium on Quantitative Biology **27**: 345–351, 1992

Hedrick L, Cho KR, Fearon ER, Wu TC, Kinzler KW and Vogelstein B (1994) The DCC gene product in cellular differentiation and colorectal tumorigenesis. *Genes Dev* **8**: 1174–1183

Hohne MW, Halatsch ME, Kahl GF and Weinel RJ (1992) Frequent loss of expression of the potential tumor suppressor gene DCC in ductal pancreatic adenocarcinoma. *Cancer Res* **52**: 2616–1619

Iacopetta B, DiGrandi S, Dix B, Haig C, Soong RA and House A (1994) Loss of heterozygosity of tumor suppressor gene loci in human colorectal carcinoma. *Eur J Cancer* **30**: 664–670

Iino H, Fukayama M, Maeda Y, Koike M, Mori T, Takahashi T, Kikuchi-Yanoshita R, Miyake M, Mizuno S and Watanabe S (1994) Molecular genetics for clinical management of colorectal carcinoma. 17p, 18q, and 22q loss of heterozygosity and decreased DCC expression are correlated with the metastatic potential. *Cancer* **73**: 1324–1331

Inokuchi K, Miyake K, Takahashi H, Dan K and Nomura T (1996) DCC expression in hematopoietic cell populations and its relation to leukemogenesis. *J Clin Inv* **97**: 852–857

Itoh F, Hinoda Y, Ohe M, Ohe Y, Ban T, Endo T, Imai K and Yachi A (1993) Decreased expression of DCC mRNA in human colorectal cancers. *Int J Cancer* **53**: 260–263

Kashiwaba M, Tamura G and Ishida M (1995) Frequent loss of heterozygosity at the deleted in colorectal carcinoma gene locus and its association with histologic phenotypes in breast carcinoma. *Virchows Archiv* **426**: 441–446

Kataoka M, Okabayashi T and Orita K (1995) Decreased expression of DCC mRNA in gastric and colorectal cancer. *Surg Today* **25**: 1001–1007

Kato M, Ito Y, Kobayashi S and Isono K (1996) Detection of DCC and Ki-ras gene alterations in colorectal carcinoma tissue as prognostic markers for liver metastatic recurrence. *Cancer* **77**: 1729–1735

Kern SE, Fearon ER, Tersmette KWF, Eeterline JP, Leppert M, Nakamura Y, White R, Vogelstein B and Hamilton SR (1989) Allelic loss in colorectal carcinoma. *JAMA* **261**: 3099–3103

Kikuchi-Yanoshita R, Konishi M, Fukunari H, Tanaka K and Miyake M (1992) Loss of expression of the DCC gene during progression of colorectal carcinomas in familial adenomatous polyposis and non-familial adenomatous polyposis patients. *Cancer Res* **52**: 3801–3803

Klingelhutz AJ, Hedrick L, Cho KR and McDougall JK (1995) The DCC gene suppresses the malignant phenotype of transformed human epithelial cells. *Oncogene* **10**: 1581–1586

Laelor KG and Narayanan R (1992) Persistent expression of the tumor suppressor gene DCC is essential for neuronal differentiation. *Cell Growth Diff* **3**: 609–616

Laemmli UK (1970) Cleavage of structural proteins during the assembly of the head of bacteriophage T4. *Nature* **227**: 680–685

Miyake S, Nagai K, Yoshino K, Oto M, Endo M and Yuasa Y (1994) Point mutations and allelic deletion of tumor suppressor gene DCC in human esophageal squamous cell carcinomas and their relation to metastasis. *Cancer Res* **54**: 3007–3010

Murty VV, Li RG, Houldsworth J, Bronson DL, Reuter VE, Bosl GJ and Chaganti RS (1994) Frequent allelic deletions and loss of expression characterize the DCC gene in male germ cell tumors. *Oncogene* **9**: 3227–3231

Nowell PC (1986) Mechanisms of tumor progression. *Cancer Res* **46**: 2203–2207

Ookawa K, Sakamoto M, Hirohashi S, Yoshida Y, Sugimura T, Terada M and Yokota J (1993) Concordant p53 and DCC alterations and allelic losses on chromosomes 13q and 14q associated with liver metastases of colorectal carcinoma. *Int J Cancer* **53**: 382–387

Sato Y, Mukai K, Watanabe S, Goto M and Shimosato Y (1986) The AmeX method: a simplified technique of tissue processing and paraffin embedding with improved preservation of antigens for immunostaining. *Am J Pathol* **125**: 431–435

Shibata D, Reale MA, Lavin P, Silverman M, Fearon ER, Steele G, Jessup JM, Loda M and Summerhayes IC (1996) The DCC protein and prognosis in colorectal cancer. *N Engl J Med* **335**: 1727–1732

Smith DB and Johnson KS (1988) Single-step purification of polypeptides expressed in *Escherichia coli* as fusions with glutathione S-transferase. *Gene* **67**: 31–40

Thiagalingam S, Lengauer C, Leach FS, Schutte M, Hahn SA, Overhauser J, Willson JK, Markowitz S, Hamilton SR, Kern SE, Kinzler KW and Vogelstein B (1996) Evaluation of candidate tumour suppressor genes on chromosome 18 in colorectal cancers. *Nature Genet* **13**: 343–346

Turley H, Pezzella F, Kocialkowski S, Comley M, Kaklamanis L, Fawcett J, Simmons D, Harris AL and Gatter KC (1995) The distribution of the deleted in colon cancer (DCC) protein in human tissues. *Cancer Res* **55**: 5628–5631

Vogelstein B, Fearon ER, Hamilton SR, Kern SE, Preisinger AC, Leppert M, Nakamura Y, White R, Smits AMM and Bos JL (1988) Genetic alterations during colorectal-tumor development. *N Engl J Med* **319**: 525–530

Yamashiro S, Ruan S, Furukawa K, Tai T, Lloyd KO, Shiku H and Furukawa K (1993) Genetic and enzymatic basis for the differential expression of GM2 and GD2 gangliosides in human cancer cell lines. *Cancer Res* **53**: 5395–5400

© Cancer Research Campaign 1998